# Coordinated modular functionality and prognostic potential of a heart failure biomarker-driven interaction network

**DOI:** 10.1186/1752-0509-4-60

**Published:** 2010-05-12

**Authors:** Francisco Azuaje, Yvan Devaux, Daniel R Wagner

**Affiliations:** 1 Laboratory of Cardiovascular Research, Centre de Recherche Public - Santé, L-1150, Luxembourg; 2 Division of Cardiology, Centre Hospitalier, L-1210, Luxembourg

## Abstract

**Background:**

The identification of potentially relevant biomarkers and a deeper understanding of molecular mechanisms related to heart failure (HF) development can be enhanced by the implementation of biological network-based analyses. To support these efforts, here we report a global network of protein-protein interactions (PPIs) relevant to HF, which was characterized through integrative bioinformatic analyses of multiple sources of "omic" information.

**Results:**

We found that the structural and functional architecture of this PPI network is highly modular. These network modules can be assigned to specialized processes, specific cellular regions and their functional roles tend to partially overlap. Our results suggest that HF biomarkers may be defined as key coordinators of intra- and inter-module communication. Putative biomarkers can, in general, be distinguished as "information traffic" mediators within this network. The top high traffic proteins are encoded by genes that are not highly differentially expressed across HF and non-HF patients. Nevertheless, we present evidence that the integration of expression patterns from high traffic genes may support accurate prediction of HF. We quantitatively demonstrate that intra- and inter-module functional activity may be controlled by a family of transcription factors known to be associated with the prevention of hypertrophy.

**Conclusion:**

The systems-driven analysis reported here provides the basis for the identification of potentially novel biomarkers and understanding HF-related mechanisms in a more comprehensive and integrated way.

## Background

Heart failure (HF) is a clinical syndrome that results from cardiac disease. HF can be characterized as the heart's inability to pump enough blood to meet physiological requirements. HF may be caused by cardiac injury (e.g. failure after myocardial infarction) or by non-ischemic diseases (e.g. dilated cardiomyopathy). Independently of the etiology, HF is known to be the by-product of a large-scale, dynamic interplay of proteins, hormones and metabolites. These interactions are in turn brought about and controlled by a diversity of genes and molecular pathways responsible for different processes, which range from inflammation trough extracellular-matrix remodeling to angiogenesis. This motivates the development of approaches to the systematic, integrated analysis of protein interactions related to HF.

Many questions connected to the elucidation of the complex molecular mechanisms spurring the emergence, progression and repair of cardiac malfunction remain to be answered. The increasing amounts of information about accepted and putative HF biomarkers and therapeutic targets, as well as of annotated datasets of protein-protein interactions (PPIs) in humans, offer new opportunities to understand HF within a systems biology framework [1].

Advances in high-throughput technologies for the quantitative assessment of different "omic" information variables are fostering a more comprehensive, systems-level view of the PPIs involved in different physiological and pathological conditions. Over the past few years, larger amounts of experimentally-validated human PPIs [1-3] have been made available via public or proprietary Web-based information resources. Advances in this area have traditionally concentrated on the analysis of large-scale, global PPI networks (i.e., interactomes) in a small number of model organisms and more recently in humans. For instance, using experimentally-validated or expert-annotated interactomes, researchers have shown how the structure and composition of PPI networks can be linked to specific biological processes, properties and clinical outcomes [4-6]. Furthermore, investigations have demonstrated how such information can be meaningfully correlated with observations and predictions at different "omic" information levels, e.g. genomic variation [6], gene expression [7,8] and standard functional annotations [9].

Major steps forward in this area have been: a) the capacity to link clusters of highly-connected proteins (commonly referred to as "modules") within these networks and specific biological processes [4,10] and b) the capacity to detect potential biomarkers, therapeutic targets or critical functional components using network topology features [5,11]. The majority of these contributions have focused on the investigation of large-scale networks that are not specific to diseases or phenotypes. Furthermore, network-based approaches have not been sufficiently investigated in the area of HF research. The potential of network-based analyses in the cardiovascular area has been previously reported in dilated cardiomyopathy (DCM) investigations [8,12]. Zhu et al. [12] integrated public gene expression data with a layered PPI network that was organised into four functional compartments: extracellular, plasma membrane, cytoplasm and nucleus. This allowed them to identify the Janus family tyrosine kinase-signal transducer and activator of transcription (Jak-STAT) signaling pathway as a potential key driver of DCM development.

Despite the potential limitations related to knowledge incompleteness and uncertainty in the network inference process, the characterization of complex biological phenomena on the basis of functional modular architectures and topological parameters present us with new opportunities to improve our understanding of the evolution, operation and possible re-engineering of these systems [6,13,14].

Here we report the analysis of a PPI network in the context of human HF and in relation to diverse, complementary resources of "omic" information. This analysis aimed to characterize potential functional and structural patterns and associations, which may explain fundamental molecular mechanisms underlying HF, as well as the role of biomarkers, from a systems biology standpoint. The practical utility and potential biomedical relevance of the outcomes of this research are two-fold. First, the products of this research can be seen as a disease-specific knowledge reference for future research. Second, we offer testable hypotheses and predictions relevant to the discovery of potential novel biomarkers or targets based on an integrated network-based methodology. We aimed to uncover potential biologically-meaningful modules and inter-module relationships. Given that protein activity is influenced by different forms of regulation and between-process relationships, we investigated structural and functional features that can characterize the "centrality" or "traffic" mediation capability of network proteins. Finally, we addressed possible intra- and inter-module regulatory control mechanisms.

## Methods

**Figure 1:**
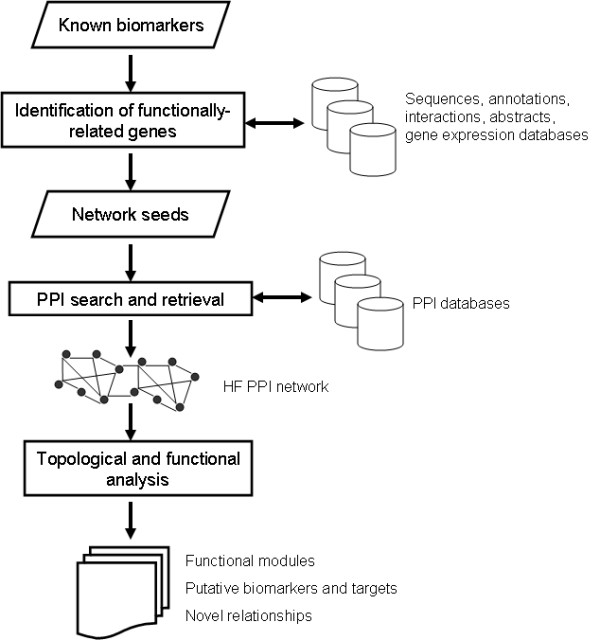
**Integrative analysis of "omic" information in the context of a HF PPI network: Overview of main analytical phases implemented in this research**. Novel relationships refer to new associations between proteins and specific processes and cellular localizations, and between functional modules and specific transcriptional regulatory mechanisms.

Figure 1 schematically summarizes the main analytical phases implemented in this research. First, a list of known HF biomarkers was gathered from the literature. This set of biomarkers was expanded by the identification of functionally-related genes using different "omic" resources and databases. The set of proteins resulting from the combination of the HF biomarkers and the retrieved functionally-related genes represented the "network seeds" in this investigation. The network seeds were used as inputs to the PPI search and retrieval phase. Using different repositories of annotated PPIs, a HF-related PPI network was assembled. Several bioinformatic analyses generated insights into structural and functional properties of this network, as well as novel associations between biomarkers and potentially-relevant network-based biological features. The latter aimed to determine potential useful relationships between the modules and different biological properties, such as specific biological processes, cellular localizations and shared transcriptional co-regulatory mechanisms within the modules.

Before providing a more detailed description of these analysis phases, basic concepts relevant to network-based biology are defined as follows.

### Basic concepts

Networks are used here to represent direct, protein-protein interactions. Network "nodes" (or "components") and "edges" (or "links") represent proteins and interactions respectively. A group of highly-interconnected nodes can be defined as a "module". A module may be identified through network clustering, which meets specific statistical analysis criteria as explained below. There are various statistical or topological features that can be used to characterize the networks, its modules, and individual components. Two such fundamental features are the node "degree" and "traffic". The degree of a node refers to the number of edges associated with a node, i.e.,, given a protein, *x*, its degree represents the number of interactions between *x *and any other proteins in the network. Nodes with large degree values are commonly referred to as "hubs". The traffic going through a network node, *x*, also referred to as its "betweeness centrality" [15,16] is a measure of the total number of shortest paths that go through *x*, and which connect any two other nodes in the network. Thus, a "traffic value" can be seen as a numerical estimation of the communication-mediating capacity of a network node, and can be used to infer "cross-communication" hotpots or "bottlenecks" in the network.

### Known biomarkers and identification of functionally-related genes

Guided by recent reviews on cardiovascular disease biomarkers [17,18], a list of known HF biomarkers was compiled as the initial set of input information to our analysis framework. At least 2 biomarkers from the following biological process categories were included, as defined in [18]: inflammation, oxidative stress, extracellular-matrix remodeling, neurohormones, myocyte injury, myocyte stress and other validated biomarkers not falling within conventional categories. Examples of biomarkers included in this list are: CRP, MPO, MMP proteins, EDN1, TNNI1, NT-proBNP and GDF15. The complete list of HF biomarkers is available in the Additional file 1 (Table S1).

Genes and proteins functionally-related to the list of known biomarkers were searched in several external databases using the Endeavour system [19]. These resources comprised annotation databases, gene sequences, public gene expression data, PPI databases, putative transcription factors binding sites, abstracts and computational predictions of gene-disease associations. The goal was to retrieve genes and proteins from those resources that were functionally "similar" to the list of known HF biomarkers. Similarity or functional relatedness was estimated by different data type-specific criteria. For example, gene-gene similarity at the DNA sequence level was estimated using BLAST searches. Correlation coefficients were used to measure similarity between genes at the expression level using data stored in public repositories. The number of Gene Ontology (GO) terms or domain family annotations shared by a pair of gene products was used to assess annotation-based functional similarity. Endeavour calculates annotation-based similarity between genes by applying the Fisher's omnibus method [19]. Given two genes with shared annotations, this technique combines the *P *values of those annotations that were found statistically-enriched in the set of known biomarkers. For each "omic" database, a list of functionally-related "candidate" genes was retrieved and ranked on the basis of their corresponding similarity scores. After performing a whole-genome search, a global ranking of candidate genes was obtained by combining all the database-specific similarity scores into a single similarity score based on order statistics, as proposed by [19]. Thus, the list of candidate genes retrieved was defined as functionally similar to the set of input HF biomarkers, as a whole. In this analysis, we focused on the top-100 genes retrieved by this procedure. These genes are those reporting the lowest ranking scores (RS) as estimated by the Endeavour system (RS < 2E-6). There is no empirical or standard approach for defining ranking thresholds using these scores. Although this score estimates the likelihood that a candidate gene would obtain the observed rank by chance, these scores cannot be interpreted as probability values. By focusing on these top 100 genes we also aimed to obtain a list of the most relevant, potentially biomarker-associated candidates, which may in turn contribute to the reduction of false positive associations. Furthermore, this selection allowed the inclusion of a list of known biomarkers, as well as a list of candidate biomarkers at least twice as large as the list of known biomarkers.

The complete list of candidate genes obtained and their similarity rankings are available in the Additional file 1 (Table S2). Additional information on the computational implementation of this search and retrieval procedure is offered below.

The combination of the list of HF biomarkers and the candidate genes defined the set of network seeds, which were used as inputs to the PPI network construction phase.

### PPI network construction

PPIs associated with the network seeds were identified and retrieved from different public databases of annotated interactions. The PPI databases included in this analysis were: the Human Protein Reference Database (HPRD) [20], the general repository for interaction datasets (BioGRID) [21], and the Molecular INTeraction database (MINT) [22]. Only expert-annotated interactions observed in humans were considered. The retrieved interactions were combined into a unique set of interacting pairs. This integrated PPI set represented the union of the database-specific sets of retrieved PPIs. The obtained PPIs were used to assemble the HF PPI network. The network consists of all interactions of the network seeds. Thus, we concentrated on first-level interacting partners. This allowed us to focus on a set of highly relevant proteins in relation to known HF biomarkers. Also we aimed to reduce the amount of potential false positive interactions in our disease-specific network. We admit that this is done perhaps at the expense of potentially novel, deeper interactions. Nevertheless, at present it would not be possible to obtain accurate, experimentally-validated interactions belonging to expanded interaction levels in the specific context of human HF.

### Detection of network modules

The resulting network was digitally encoded, displayed and analyzed using different topological and functional features. Potential network modules were identified with a "greedy" network clustering algorithm [23]. This algorithm searches for network regions with highly-connected nodes, and the outcome is the partition of the network into a set of disjoint clusters that optimize a "modularity score", *Q*. The *Q *score measures the number of edges observed in a putative module in relation to the number of edges that would be obtained by randomly pairing the proteins in the module [16,23]. The *Q *score is defined as [16]:

Where *numIME *is the number of intra-module edges, and *numE *is the total number of network edges. The first term deals with the network investigated, while the second refers to a randomized version of the network: nodes are randomly connected with node degree preservation.

Thus, the module discovery algorithm searches for groups of inter-connected nodes that maximize the *Q *score. This algorithm has allowed the identification of biologically-relevant modules, such as protein complexes and other functionally-related protein clusters, in different model organisms and humans [4,5,10,24].

### Gene expression analyses

To assess the potential application of the network-based predictions of putative biomarkers or targets, we analyzed their gene expression in the context of two groups of patients after acute myocardial infarction (MI): a group of 16 patients with HF, and a group of 16 non-HF patients. The non-HF patients exhibited preserved left ventricular (LV) systolic function and high ejection fraction (EF) after MI (EF > 40%, median 63%, range 45-73). The HF group presented impaired LV function and low EF (EF ≤ 40%, median 35%, range 20-40). Patient characteristics are gathered in Table S3 (Supplementary Section).

Acute MI was defined by the presence of chest pain <12 hours with significant ST elevation and increase in creatine kinase and troponin I to greater than 2-fold upper limit of normal levels. Blood samples were obtained at the time of mechanical reperfusion. EF was determined by echocardiography 1 month after MI. All patients signed an informed consent.

Total RNA was extracted from 2.5 mL of whole blood by the PAXgene™ technology. Gene expression profiles of blood cells were obtained with genome-wide arrays [25].

### Ethics Statement

The protocol was approved by the local ethics committees (Comité national d'éthique de la recherche, CNER; Comission nationale pour la protection des données, CNPD) and written informed consent was obtained from all patients.

### Software tools, additional information resources and basic statistical analyses

The identification of genes functionally-related to the set of known HF biomarkers was implemented with the ENDEAVOUR system [26]. Only functional relationships involving human genes/proteins were considered. The list of HF biomarkers was used as the set of "training" genes. All "omic" data sources integrated under ENDEAVOUR were selected to search for the candidate genes, as defined above. PPIs were searched and retrieved with in-house developed software coded in Java (Additional file 1). Exploratory network visualization and exploration tasks were performed with Cytoscape [27] and Polar Mapper [16].

Network module identification and traffic estimations were carried out with Polar Mapper [16]. Network modules were characterized on the basis of GO biological process and cellular localization terms that were statistically over-represented in the modules. This was implemented with the Fatigo tool, under the BABELOMICS (v3.1) software platform [28]. *P *values describing the statistical strength of these associations were estimated with (two-tailed) Fisher's exact tests and corrected to account for multiple-hypotheses testing using the Benjamini & Hochberg adjustment procedure [28]. Unless otherwise indicated, only corrected *P *values are reported here and statistical significance is defined at the *P *= 0.05 level. Other GO annotation visualization tasks were carried out with GenNav v1.11 [29]. Whole-genome associations between transcription factors (TFs) and microRNAs (miRNA) and the genes defining the network modules were estimated using the same statistical testing procedures and software tool. TF- and miRNA-module associations were searched in the TRANSFAC [29] and miRBase [31] databases respectively.

Independent, two-group statistical comparisons were performed with the Student's *t*-test. Correlation between numerical variables was estimated with the Pearson's correlation coefficient. These calculations and statistical displays were implemented with the *Statistica *package [32] and T-REX under the GEPAS 4.0 platform [33]. Classification models based on gene expression data were implemented with Weka [34]. Other network-based statistical measures were computed with (Java-written) software developed in our laboratory.

## Results

### Known biomarkers and network protein seeds

The set of known HF biomarkers included 37 proteins (Table S1). Out of the top 100 genes that were found to be functionally related to the set of known biomarkers as a whole, 32 genes actually encoded known protein biomarkers. Thus, a total of 68 (unique) genes were found to be functionally relevant to the set of known biomarkers. The union of the set of known biomarkers and their functionally-related genes represented the "network seeds". The network seeds comprised a total of 105 proteins (Tables S1 and S2), and were used to assemble the PPI network as described in the Methods section.

### A core network of PPIs in HF

**Figure 2:**
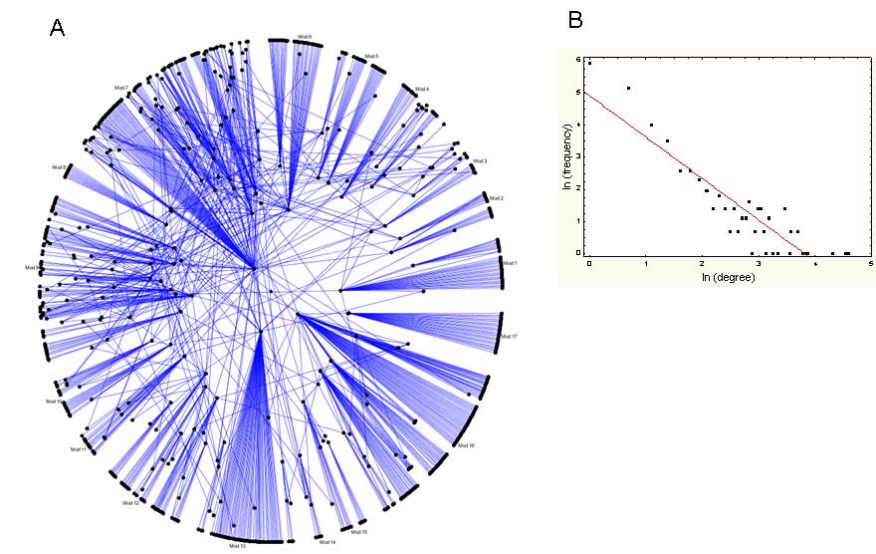
**Schematic representations of PPI networks related to HF**. A. Core HF PPI network. B. Degree distribution of the core network. In A, nodes and edges represent proteins and interactions respectively. Nodes located near the center represent highly-connected nodes, or nodes at the intersection between different node-node pathways. In B, the relationship between node degree and the observed frequency is plotted on a logarithmic scale (ln) for this network.

The PPI search and retrieval task generated a global network consisting of 772 nodes (proteins) and 1443 edges (PPIs). The resulting network comprises a single "core network" (Figure 2A), which is composed of 746 proteins and 1420 interactions. This is the single, largest interconnected region of the global network. In addition, six small "islands" of interconnected proteins detached from the core network were found. These islands included from 2 to 8 proteins, and from 2 and 14 interactions (not shown). Figure 2 also shows the degree distribution of the core network. The global and core networks follow a power-law degree distribution of the form: *D*(*k*)~*k*^-γ^, where *k *represents the degree values observed in the network, *D*(*k*) is the observed frequency of nodes with degree *k*, γ is known as the power-law exponent and ~ represents proportionality between these variables. On a logarithmic scale, as shown in Figure 2B, the power-law exponent, γ, represents the slope of the line fitted to this distribution. In this analysis the global and core networks exhibited a γ = 1.29. In previous research, power-law distributions have been used to characterize different biological networks, and they reflect the diversity of the number of connections exhibited by the nodes in a network. A power-law distribution indicates that the majority of the network nodes tend to show small numbers of interactions and that only a minority of nodes is highly-connected.

Hereafter we focus our analyses on the core network. The core network not only concentrates the vast majority of nodes and edges, but also represents a single inter-connected network that preserves the degree distributions of the "global" set of nodes and interactions. By focusing on the core network, we also aim to identify biologically-meaningful interrelated groups of proteins based on the structural analysis of this network, e.g. detection of modules of inter-connected nodes.

### Functional landscape and modular organization of the HF PPI network

The clustering analysis of the core network generated 17 clusters of highly-connected proteins, which are hypothesized as potential functional modules in this network. Table 1 describes major features of these modules, including GO biological processes and cellular localizations statistically over-represented in each module. Such statistical estimations were made after correcting *P *values for multiple-testing. The vast majority of modules can be labeled with a variety of GO biological processes (16 out of 17 modules), and 14 of these modules can be assigned with confidence to specific cellular compartments. Module 17 did not show statistical enrichment of GO terms after making multiple-testing adjustments. Modules 10 and 14 did not include over-represented GO cellular localization terms at the nominal (*P *= 0.05) level. Overall, many of these modules tend to be implicated in cell adhesion and immune responses, with a significant number of proteins expressed in extracellular regions. However, a diverse range of specialized functional roles and cellular localizations were also observed. Examples of the former include: muscle contraction, tissue remodeling and signal transduction. Moreover, partial functional overlaps were observed. A more detailed description of these modules is available in the Additional file 1 (Table S4).

**Table 1:** Overview of functional modules indentified through network structural analysis.

Network module	Number of proteins	Number of interactions	Median traffic	Number of IMIs	BP	CC
1	33	87	64	2	Innate immune response (*P *= 2.0E-22)	Extracellular region part (*P *= 5.1E-17)

2	17	57	32	3	Immune response (*P *= 5.0E-04)	Cell part (*P *= 0.01)

3	21	84	40	12	Cell adhesion (*P *= 1.8E-06)	Extracellular matrix part (*P *= 8.4E-14)

4	33	84	64	7	Circulation (*P *= 2.3E-19)	Plasma membrane (*P *= 0.003)

5	32	94	62	7	Coagulation (*P *= 1.6E-08)	Extracellular region part (*P *= 2.0E-04)

6	37	117	72	4	Tissue remodeling (*P *= 8.4E-05)	Integral to plasma membrane (*P *= 0.005)

7	133	731	264	46	Cell adhesion (*P *= 2.7E-18)	Extracellular region part (*P *= 9.9E-47)

8	11	26	20	1	Taxis (*P *= 1.9E-14)	Plasma membrane part (*P *= 5.4E-06)

9	100	469	198	31	Protein digestion (*P *= 2.6E-13)	Extracellular region part (*P *= 5.1E-33)

10	24	64	46	7	Positive regulation of signal transduction (*P *= 1.3E-05)	?

11	19	54	36	8	Proteolysis (*P *= 2.4E-13)	Endoplasmatic reticulum (*P *= 0.004)

12	51	182	100	8	Immune response (*P *= 7.1E-13)	Extracellular space (*P *= 0.002)

13	68	212	134	14	Cell adhesion (*P *= 4.4E-15)	Receptor complex (*P *= 7.5E-12)

14	7	21	12	3	Cell adhesion (*P *= 2.0E-04)	?

15	18	42	34	4	Muscle contraction (*P *= 5.1E-05)	Myofibril (*P *= 9.0E-04)

16	112	390	222	9	Cell communication (*P *= 7.7E-27)	Cytoplasm (*P *= 0.007)

17	30	91	58	1	Response to stress* (*P *= 0.01)	DNA-directed RNA polymerase complex* (*P *= 0.04)

BP and CC are examples of biological processes and cellular localizations terms respectively, which are highly over-represented in each module, as defined in the GO. *P *values estimating the statistical significance, after correcting for multiple-testing, of the enrichment of the terms are also included. "*": enrichment of GO term in the module was nominally significant at *P *= 0.05, i.e., no statistical significance was observed after correcting *P *value for multiple-testing. "?": statistically detectable GO terms were not found. The numbers of interactions includes both inter- and intra-module interactions. Additional details are included in the Additional file 1 (Table S4). BP: biological process. CC: cellular component. IMIs: Inter-module interactions.

**Figure 3:**
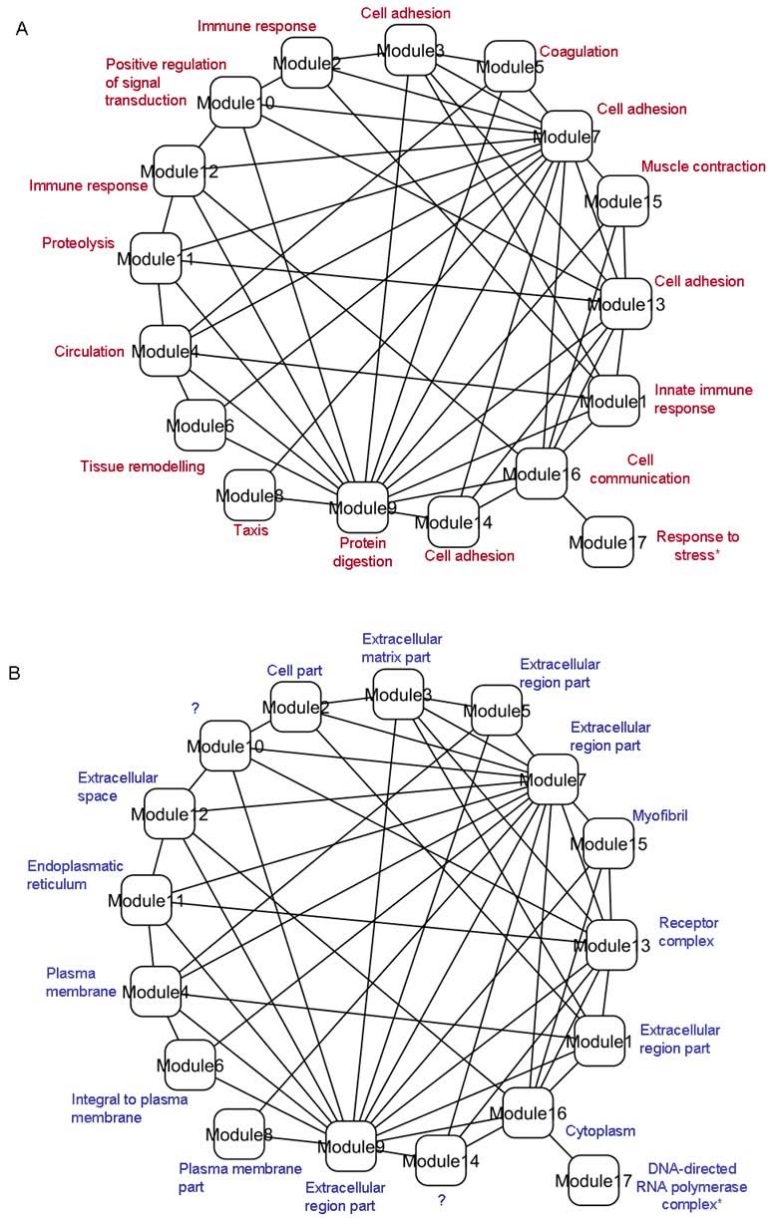
**Functional landscape and module-oriented architecture of the HF PPI network**. Lines linking the modules represent inter-module interactions (independently of the number of individual PPIs). A. Functional characterization based on module-specific involvement in different biological processes. B. Functional characterization based on module-specific associations with cellular localizations.

These structural and functional features provide evidence of a hierarchical, module-oriented organization of the HF biomarker-centric network investigated here. This allows one to visualize their structure and functionality at a higher level of complexity, in which highly specialized, yet partially overlapping, functional modules interact and orchestrate their functional contributions to processes implicated in HF. Figure 3 graphically illustrates this functional landscape and module-oriented architecture. Lines linking the different modules represent inter-module PPIs. Figures 3A and 3B describe each module on the basis of statistically representative GO biological processes and cellular localizations, as described in Table 1. A more detailed description of biological processes and cellular localizations is given in the Additional file 1 (Table S4). This visualization reveals the existence of strong interplay and cross-communication between the different functional modules. Moreover, it indicates that modules not only tend to interrelate to other modules involved in similar processes, but also to modules implicated in dissimilar or complementary roles. For example, modules specialized in cell adhesion tend to interact with each other, as in the case of Modules 7 and 2, but also with modules strongly involved in muscle contraction and tissue remodeling. Figure 3B also illustrates the coordinated interplay between modules across different cellular regions, e.g. cytoplasm-extracellular space and organelle-membrane interactions.

A GO-based functional analysis of the set of known biomarkers reported the following biological processes as statistically detectable (strongest enrichments below level 3 of GO): blood pressure regulation (*P* = 1.35E-05), organismal catabolic process (*P* = 1.35E-05) and protein digestion (*P* = 1.35E-05). In addition, the extracellular space was the localization with the most significant enrichment of biomarkers (*P* = 1.09E-16). The module-based analysis detected significant associations that were not found in the list of biomarkers, e.g., tissue remodelling (Module 6, *P* = 8.4E-05). We also observed that the statistical significance of the biological process terms overrepresented in the set of known biomarkers was weaker than that observed in the module-based analysis. For example, "blood pressure regulation" (*P* = 1.35E-05) was the most over-represented biological process in the set of biomarkers. In the network-based analysis, the enrichment of this term (Module 4) was statistically detectable at *P* = 3.33E-11. This indicates that our network-based approach was capable to recognise known relationships, as well as more specific ones, which could not have been detected by solely looking into the list of known biomarkers.

### Inter-module relationships and network highways

These modules can also be characterized on the basis of their cross-communication capability. This is important to identify potential communication "coordination" or "routing" centers, which may play major roles in the regulation of functional activity at a systems-level. The modules incorporate different numbers of inter-module interactions, ranging from 1 (Module 17) to 46 (Module 7) (Table 1). Aside from Module 7, Modules 9 and 13 represent examples of major routing centers of information flow in this network, with 31 and 14 inter-module interactions respectively. Module 7 is the largest network module and is enriched in proteins heavily implicated in diverse processes, such as cell adhesion (*P *= 2.7E-18), anatomical structure development (*P *= 9.8E-09) and coagulation (*P *= 2.4E-07). This module appears to mainly operate in the extracellular region part (*P *= 9.9E-47) and collagen (*P *= 6.9E-33), as defined by the GO.

Based on an estimation of "traffic" values (Table 1), Module 7 again is shown to be the module with the strongest inter-module mediating capability (median traffic = 264). This indicates that Module 7 represents an efficient (or short) communication pathway to link different proteins and modules across the different locations in this network. For all the network modules, the number of inter-module interactions is strongly correlated with their respective (median) traffic values (Pearson correlation: *r *= 0.81, *P *= 0.0007).

### Network hubs tend to be high-traffic nodes

**Figure 4:**
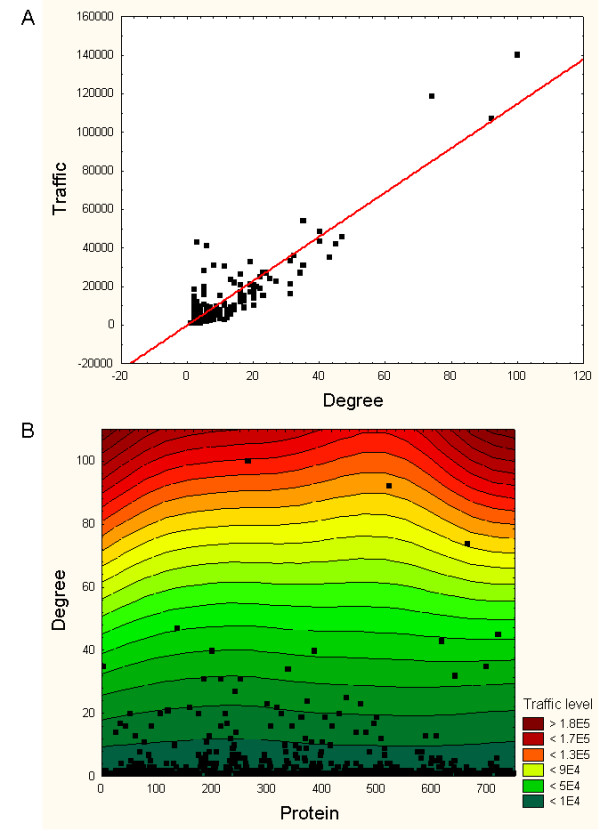
**Characterization of major network communication properties**. A. Relationship between node degree and traffic. B. A 3D contour plot of the communication and connectivity structure of the HF network. In A. a line is fitted to the data to highlight the linear relationship between the variables. In B. the black squares represent network proteins plotted against their corresponding degree values, and the colour-coded regions reflect the traffic levels. Colour regions and contours were fitted according to a distance weighted least squares procedure. The higher the position of a protein on the plot, the larger its number of connections and traffic level. Fibronectin 1 (FN1), integrin beta 1 (ITGB1) and platelet-derived growth factor receptor beta (PDGFRB) are the top three communication "hotpots" with the highest degree and traffic values in this network.

We found a strong linear correlation between node degree and traffic (*r *= 0.92, *P *= 0.0001). Figure 4A illustrates this relation for all the core network nodes. An alternative view of the underlying communication and connectivity structure of the HF network is shown in Figure 4B. In this contour plot, the black squares represent network proteins plotted against their corresponding degree values, and the color-coded regions reflect the traffic levels for these proteins. The higher the position of a protein on the plot, the larger their degrees and traffic levels. Thus, red regions reflect the existence of network "superhighway" hotpots, i.e., those nodes with both high traffic levels and number of connections. Proteins fibronectin 1 (FN1), integrin beta 1 (ITGB1) and platelet-derived growth factor receptor beta (PDGFRB) represent the top three 3 communication "hotpots" with the highest degree and traffic values in this network. Such superhighway nodes not only represent central components with strong influence in the structural integrity of the network. They also define key mediating nodes in the network, i.e., they facilitate closer interactions between different modules and individual proteins by providing shorter communication pathways between them.

### Known biomarkers tend to be hubs and high-traffic hotpots in the HF network

A comparison between the group of known HF biomarkers and the other nodes in the network showed statistically detectable differences in terms of node degrees and traffic levels. Known biomarkers, in average, have more connections (*t *= 6.60, *P *= 1E-07) and tend to exhibit greater traffic levels (*t *= 7.85, *P *= 1E-07) in comparison to the other proteins included in the core network.

### Prediction of putative novel biomarkers and targets

Based on the evidence that HF biomarkers tend to be high-traffic nodes, we suggest that such a network-based feature may be used to point out to potential new biomarkers or targets. Table 2 displays the top-20, high-traffic proteins in the HF core network together with representative GO annotations and their network module locations. The top five, high-traffic proteins are FN1, PDGFRB, ITGB1, complement component 3 (C3), collagen type I alpha 1 (COL1A1) and transforming growth factor beta 1 (TGFB1) (also see Figure 4). Among the list of proteins with known associations with HF, the following proteins were included in this top ranking: matrix metalloproteinase 2 (MMP2), chromogranin B (CHGB), collagen type II alpha 1 (COL2A1), tumor necrosis factor (ligand) superfamily member 11 (TNFSF11), matrix metalloproteinase 9 (MMP9) and tumor necrosis factor (TNF). A diversity of biological processes, ranging from cell adhesion to G-protein coupled receptor protein signaling pathways, can be found in this ranking. Moreover, these proteins have been assigned to different cellular compartments, including cytoplasm, plasma membrane and different protein complexes. Table 2 also shows distinguishing features between HF biomarkers and other high-traffic proteins. Proteins from the latter group are annotated to GO biological processes that are not observed in the annotation set of (high-traffic) HF biomarkers, such as proteins with angiogenic and metabolic roles. Examples are COL1A1 (blood vessel development), LRP1 (lipid metabolic process) and PDGFRB (positive regulation of cell proliferation). The correlation between the top-20 high-traffic proteins and the top-20 most-connected proteins was: 0.98 (Pearson correlation, *P *= < 1E-5) and 0.84 (*P *= <1E-6) for traffic and degree values respectively.

**Table 2:** Top-20 high-traffic proteins in the HF core network.

Protein	Network module	BP	CC	Traffic
FN1	7	cell adhesion, response to wounding	extracellular region	140037.9

PDGFRB	16	positive regulation of cell proliferation, positive regulation of cell migration	?	118789.9

ITGB1	13	homophilic cell adhesion, leukocyte adhesion	cell surface	107208.2

C3	1	G-protein coupled receptor protein signaling pathway	extracellular region	53979.1

COL1A1	7	blood vessel development	plasma membrane	48837.3

TGFB1	6	connective tissue replacement during inflammatory response	extracellular region	45861.8

**MMP2**	**9**	**proteolysis**	**extracellular space**	**43355.8**

PTEN	17	regulation of cyclin-dependent protein kinase activity	cytoplasm	43018.0

**CHGB**	**17**	**?**	**?**	**42398.0**

ITGB3	7	blood coagulation	integrin complex	41256.5

ADAM15	16	cell-matrix adhesion	?	36080.8

IL6ST	16	positive regulation of cardiac muscle hypertrophy	interleukin-6 receptor complex	35295.1

**COL2A1**	**7**	**collagen fibril organization**	**collagen type II**	**33618.7**

**TNFSF11**	**9**	**immune response**	**extracellular region**	**32703.6**

ITGAV	9	cell adhesion	cytoplasm	31291.3

PDGFRA	16	cell activation	integral to plasma membrane	31130.0

LRP1	9	lipid metabolic process	membrane fraction	30483.8

SRC	16	protein kinase cascade	plasma membrane	28672.6

**MMP9**	**9**	**macrophage differentiation**	**extracellular space**	**27463.8**

**TNF**	**12**	**anti-apoptosis**	**plasma membrane**	**27462.7**

Bold rows highlight proteins known to be HF biomarkers. For each protein, BP and CC represent examples of biological processes and cellular localization annotations respectively assigned to the protein, as defined in the GO. "?" means that no GO annotation was found for the protein (as of January 2009).

### Gene expression of putative biomarkers and targets

A traditional approach to identify potential biomarkers or targets is to estimate the differential expression of genes across clinically-relevant groups of samples. Using microarray technology, we analyzed the expression of the genes encoding the top-20 high-traffic proteins across HF and non-HF samples, as specified in Methods. Statistical analyses (*t-*tests) reported very low differential expression of these genes between the clinical classes (Table 3). No statistically detectable differences were observed at (or around) the *P *= 0.05 level, except in the cases of TGFB1 (*t *= -1.98, *P *= 0.06) and PTEN (*t *= -2.45, *P *= 0.02). Traffic values and levels of differential expression (as estimated by the *t-*statistic) are indeed uncorrelated (Spearman correlation, *r *= 0.09). This shows how these potentially influential proteins could not have been detected on the basis of their gene expression alone. However, the potential of gene expression information of high-traffic proteins for biomarker or therapeutic target applications deserves further investigations. We found that patient classification models built on the expression values of TGFB1 and PTEN could correctly classify 72% of these samples using a linear support vector machine classifier (SVM) and leave-one-out cross-validation (LOO). To further explore the potential prognostic accuracy of these genes, we implemented SVM classifiers using high-traffic, known biomarkers as inputs (Table 1). Different models based on the combination of the expression values of these biomarkers reported classification performances equal or worse than random prediction (LOO, area under the receiver operating characteristic curve, AUC < 0.5).

**Table 3:** Differential gene expression of the top-20 high-traffic proteins between HF and non-HF patients.

Protein	*t*-statistic	*P*	Traffic
FN1	-0.99	NS	140037.9

PDGFRB	0.62	NS	118789.9

ITGB1	-0.44	NS	107208.2

C3	1.12	NS	53979.1

COL1A1	1.43	NS	48837.3

TGFB1	-1.98	0.06	45861.8

**MMP2**	**0.16**	**NS**	**43355.8**

PTEN	-2.45	0.02	43018.0

**CHGB**	**0.27**	**NS**	**42398.0**

ITGB3	NA	NA	41256.5

ADAM15	-0.21	NS	36080.8

IL6ST	NA	NA	35295.1

**COL2A1**	**NA**	**NA**	**33618.7**

**TNFSF11**	**NA**	**NA**	**32703.6**

ITGAV	NA	NA	31291.3

PDGFRA	-0.65	NS	31130.0

LRP1	-0.37	NS	30483.8

SRC	-0.54	NS	28672.6

**MMP9**	**-1.15**	**NS**	**27463.8**

**TNF**	**1.39**	**NS**	**27462.7**

Bold rows highlight known HF biomarkers. *t*-statistic and *P *values of differential expression between HF and non-HF patients: 16 samples in each class. NS: Non-significant difference or difference statistically detectable at *P *> 0.1. 'NA': data not available in our expression dataset. A negative *t*-statistic means that the gene has a higher expression in the HF class.

### Functional modules can be regulated at the transcriptional level

**Figure 5:**
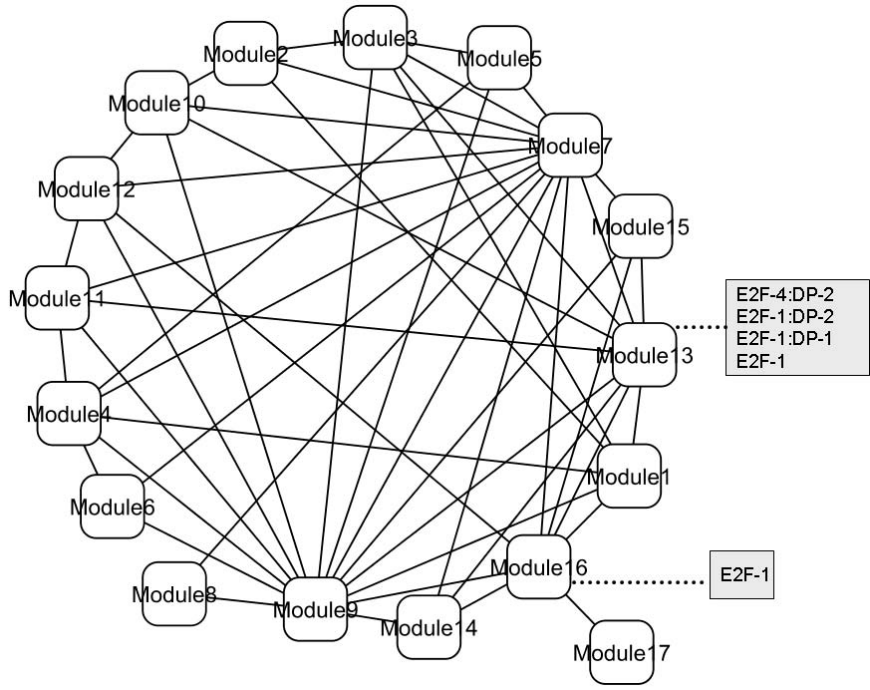
**Regulation of modules at the transcriptional level through the action of transcription factors with known strong associations with module members**.

A closer look at the composition of the network modules gives evidence of the existence of shared regulatory mechanisms at the transcriptional level. We investigated possible regulatory mechanisms through an analysis of statistical associations between these modules and known transcription factors and miRNAs. Figure 5 depicts the statistically detectable associations observed between transcription factors and functional modules. No statistical associations between known miRNAs and these modules were found at the (nominal) *P *= 0.05 level. All the transcription factors belong to the E2F family of transcription factors. This family is known to regulate cell proliferation and tumor suppressor proteins, and to be a target of small DNA tumor viruses [35,36].

Table 4 offers a more detailed description of these associations, including examples of GO biological process over-represented in the target module (statistically detectable at *P *< 0.05, after adjusting for multiple-testing). Table 4 also shows the statistical strength, *P*_*TF-Module*_, of the associations between individual transcription factors and modules. Muscle creatine kinase (CKM) and interleukin 6 (IL6) are examples of HF biomarkers included in the regulated modules. ITGB1, four and a half LIM domains 2 (FHL2), collagen type VIII alpha 1 (COL8A1), CKM, and integrin alpha 2 (ITGA2) represent the highest traffic nodes controlled by the E2F family.

**Table 4:** Transcription factors (TFs) strongly implicated in the regulation of HF core network modules.

TFs	Module	BP	***P***_***TF-Module***_	KHFB	HTraffic
					ITGB1
					FHL2
E2F-4:DP-2	13	Cell adhesion, signal transduction	0.04	CKM	COL8A1
					CKM
					ITGA2

					ITGB1
					FHL2
E2F-1:DP-2	13	Cell adhesion, signal transduction	0.04	CKM	COL8A1
					CKM
					ITGA2

					ITGB1
					FHL2
E2F-1:DP-1	13	Cell adhesion, signal transduction	0.04	CKM	COL8A1
					CKM
					ITGA2

					ITGB1
			0.04	CKM	FHL2
E2F-1	13	Cell adhesion, signal transduction			COL8A1
					CKM
					ITGA2

					PDGFRB
					ADAM15
E2F-1	16	Cell communication, phosporilation	0.02	IL6	IL6ST
					PDGFRA
					GRB2

BP: Examples of GO biological process over-represented in a module (with *P *< 0.05 after adjusting for multiple-testing). *P*_*TF-Module*_: Probability value for the association TF-module (after correcting for multiple-testing). KHFB: Known HF biomarkers controlled by the TF within the specified module. HTraffic: examples of high-traffic proteins, including putative biomarkers, regulated by the specified TF.

## Discussion and conclusion

### New insights and potential implications

This research shows how a biomarker-centric interactome related to HF exhibits a functional architecture of well-specified interconnected modules, which tend to be distributed across different cellular compartments. Inter-module communication is mediated by high-traffic or central proteins, which establish relatively short interaction paths between other proteins. Such a functional modularity and inter-module interplay suggest a coordinated, yet sometimes redundant, functional co-operation of processes and proteins relevant to the emergence and development of human HF. This coordinated cross-communication tends to be established by proteins that represent both high-degree and high-traffic network nodes. Moreover, these inter-module mediators appear to be responsible for linking, and possibly coordinating, the activities of multiple network modules associated with diverse functional processes and cellular localizations.

Within the proposed methodological framework and disease investigated, the systems-level analysis is promising because it can detect potentially novel genes beyond the traditional differential gene expression analysis. Our results indeed proved that this was the case by offering potentially novel biomarkers that were not only biomedically-meaningful, but also capable to enable relatively accurate patient classification.

Our results indicated that HF biomarkers implicated in different biological processes tend to represent high-traffic network nodes. High-traffic nodes can also be seen as bottlenecks or cross-talking hotspots because they represent components inter-linking different network regions and proteins. High-traffic nodes are found in a significant number of shortest paths connecting different network nodes. Previous studies have correlated high-traffic proteins with cell essentiality, pleiotropy and mediating communication roles between biological processes [11,37].

We also determined different proteins that deserve further research as potential novel HF biomarkers or therapeutic targets. These proteins have been shown to be involved in a wide range of processes, including blood vessel development and regulation of protein kinase activity. ITGB1 and FN1 were identified among the top-3 high-traffic proteins. Note that the rankings of ITGB1 and FN1 in the Endeavour-based prioritization were 66^th ^and 29^th ^respectively. Interestingly, ITGB1 is a receptor for FN1 and this ligand-receptor couple regulates leukocyte adhesion. Taking into consideration that inflammation, mediated by recruitment of circulating leukocytes to the heart through adhesion to the vessel wall, is an essential component of the pathogenesis of HF, our results suggest that ITGB1 and FN1 play key roles in the development and progression of HF. Indeed, accumulation of extracellular matrix proteins, such as laminin and fibronectin, is a hallmark of the development of HF [38], and ITGB1 protects the heart from ventricular dysfunction and failure [39]. In addition, we found a statistically significant association between module 13, to which ITGB1 belongs, and the E2F family of transcription factors. In the cardiovascular disease domain, E2F transcription factors have been linked to blood pressure regulation [40] and the prevention of the development of hypertrophy [41].

FN1 is an acute-phase reactant synthesized by hepatocytes following injury, such as MI or ischemic stroke. This may explain why transcriptomic analysis of blood cells performed in the present study failed to readily detect FN1 expression and a potential association with clinical outcome of MI patients (i.e., FN1 is not differentially expressed by HF and non-HF patients). Since FN1 does not belong to the family of known biomarkers of HF and is released in the plasma following injury, it will certainly be interesting to determine its prognostic performance and therapeutic utility in the context of HF.

Matrix metalloproteinases (MMPs) constitute a family of matrix degrading enzymes that contribute to the development of HF [42]. In the present study, two main MMP family members, MMP2 and MMP9, were found to be in the top-20 high-traffic proteins of the HF core network. In accordance with these findings, several groups including ours demonstrated the potential of MMP9 as a biomarker of HF [43-47].

Here we put forward a hypothesis about why biomarkers tend to reflect relevant states in the emergence or progression of the disease. Biomarkers can mirror inter-module and global functional activities in an integrated, network-oriented fashion. We showed how the role of potential biomarkers or therapeutic targets may be explained on the basis of network architecture features. We found that potentially important proteins are encoded by genes with relative low differential expression between HF and non-HF samples (e.g. ITGB1 and FN1). Thus, these key proteins could not have been detected using standard gene expression analysis alone because of the subtle differences in expression levels between these prognostic classes. This underscores the importance of alternative, post-genomic views of the biomarker and target discovery process based on the analysis of multiple resources of "omic" information. Previous research has shown that high-degree and high-traffic network nodes may be encoded by genes that are not necessarily highly expressed or differentially expressed across case-control samples in different diseases [7,8]. This reinforces the motivation for formulating advanced computational biomarker discovery approaches, which integrate different levels of "omic" complexity. Furthermore, we provided suggestive evidence of the potential of high-traffic proteins for patient classification using gene expression data.

The lack of differential gene expression of the known biomarkers may be explained by the following reasons:

1. Changes in the expression of these biomarkers do not necessarily rely on transcriptional modifications. Post-transcriptional and translational modification mechanisms may be also responsible for their differential expression. Many of these biomarkers are known to be differentially regulated at the protein expression level.

2. Strong differential gene expression may not be possible to detect due to the small size of our dataset. Also note that the new putative biomarkers have levels of differential expressions that are not strong enough to have allowed their discovery through standard gene expression analysis alone.

This motivated our systems-based discovery framework based on the integration of gene expression, protein-protein interactions, biological process annotations and DNA-gene interaction information.

This investigation also suggests that intra-module functional activity may be regulated by a family of transcription factors previously associated with cancer progression, blood pressure regulation and the prevention of hypertrophy [19,35,40]. Moreover, because of the inter-connected modular architecture underpinning the HF-related network studied here, one may argue that common transcriptional control may be achieved globally on different modules based on this or other sets of transcription factors. These insights offer evidence about potential therapeutic targets, which will require further computational and experimental investigations. In addition, the availability of new information sources, such as validated miRNA-target associations of higher genome-coverage, will open new research directions in this area.

### Possible limitations and future work

Key limitations of this research relate to information incompleteness and uncertainty. This investigation is based on a sample of current knowledge about HF biomarkers and the human interactome. Both information sources may represent either biased or incomplete information. Although we aimed to include a relatively large number of known biomarkers and functionally-related proteins to assemble the network, it is evident that false negative and false positive relationships may have influenced our findings. Nevertheless, because we do not use network topology information to make *de novo *predictions of interactions or functional annotations for specific proteins, we expect this potential deficiency to have a less significant effect on our findings. Our main research objective was to establish system-level associations and statistically detectable functional patterns, which may be used to characterize PPIs and processes relevant to HF. In addition, our analysis was not based on prior hypotheses or assumptions about the structure of the network. To reduce the potential number of false-positive interactions, we also focused on human-specific, experimentally-validated interactions. The strong statistical relationships found between network modules and known functional processes also suggest that the possible inclusion of false-positives interactions, as well as the possible exclusion of important biomarker-related proteins, would not represent a major drawback of this network.

The notion that known biomarkers tend to be relatively more studied than other proteins may suggest a potential source of bias in our estimations of the correlation between the traffic and degree values of the biomarkers. However, it is important to stress that in the list of known biomarkers, there are proteins that exhibit high traffic values despite their relative low degrees. The biomarkers IL6 and CHGB illustrate this scenario. On one side, IL6 exhibits a traffic level higher than that obtained for CHGB. On the other side, CHGB has more interacting partners than IL6 (45 vs. 6 connections). Furthermore, only 6 out of the 20 highest-traffic proteins in the global network originate from the list of known biomarkers. These observations indicate that the potential bias toward well-studied proteins did not significantly impact our findings.

In any case, we cannot rule out the possibility that a relative high number of false-negatives, i.e., true interactions or proteins relevant to HF that were not included in the network, may have influenced or biased the results reported here. In the long-term, the generation of more detailed descriptions of PPIs and the incorporation of emerging biomarkers, can provide conclusive confirmation or refutation of the results presented here. Future research should also incorporate additional information on biological context, such as tissue specificity or binding affinities.

Another limitation relates to the transcriptomic analysis of blood cells from MI patients. First, this analysis included only a low number of MI patients and it will be necessary to validate the gene expression data in a larger population. Second, the use of readily available blood cells instead of cardiac biopsy to draw a biosignature of HF can be questionable. However, several studies have demonstrated the usefulness of blood cell transcriptomes to identify biomarkers in both the oncology [48,49] and cardiovascular fields [50].

Only GO biological process and cellular localization annotations were examined to describe modules. This was done to emphasize the involvement of genes in entire, well-defined biological processes rather than in unique "activity" terms, as defined in the molecular function hierarchy (MF). Unlike MF terms, biological process terms can define involvement in more than one activity. In addition, MF terms tend to be good estimators of cellular localization terms, as reported in empirical studies [51]. Moreover, according to the GO consortium website, one can assume that for each biological process term there will be a corresponding MF term.

We also acknowledge that the biological relevance of network concepts, such as betweenness centrality or traffic, and their direct correspondence with biological information transfer phenomena remains a topic of discussion. Despite the need for additional research in this area, network-oriented concepts have proven to be powerful approaches to the generation of testable predictions about the functionality of complex biological systems in different model organisms and diseases [5,19,50,52-54].

Key questions that deserve to be investigated are: What is the effect of incorporating multi-level interaction networks (i.e. additional interactions moving away from the first-level of biomarker-protein interactions)?; how the HF-specific network relates to other phenotype-specific networks?; what place the HF-specific network occupies in a hierarchical organisation of the human interactome?; how such knowledge may facilitate the discovery of new key mediators of HF?. Answers to these questions will require larger, high-quality annotated PPI networks, with proven implications in human heart failure. Another relevant topic is the role of network-based differential expression for prognostic applications, as investigated in [55] for breast cancer. In a paper published elsewhere, we reported an alternative approach in the specific domain of ventricular dysfunction [56].

### Summary of main findings

The main findings of this investigation can be summarized as follows:

1. Well-defined functional modularity characterizes a HF biomarker-driven PPI network. Such modules can be assigned to specific cellular regions and their functional roles tend to partially overlap.

2. Inter-module relationships are established and facilitated by nodes exhibiting high-traffic or centrality betweenness in the network.

3. HF biomarkers tend to represent network hubs and high-traffic nodes in this network.

4. Putative biomarkers or targets may be predicted on the basis of their traffic level properties.

5. High traffic proteins may be encoded by genes with relatively low differential expression between HF and non-HF in post-MI patients.

6. The integration of expression data from high-traffic genes may provide the basis for potentially accurate prognostic systems.

7. Intra- and inter-module functional activity may be regulated by a family of transcription factors known to be implicated in cancer progression, blood pressure regulation and the prevention of hypertrophy.

In conclusion, this investigation contributes to the development of new global insights into complex molecular interactions and processes relevant to human HF. These results offer alternative views of crucial mechanisms driving biomarker-related functionality and organization at a systems level. Based on our analyses, HF biomarkers may be characterized as key coordinators of intra- and inter-module communication.

## Authors' contributions

FA conceived the integrated approach, implemented the computational methodology and wrote the manuscript. YD and DW provided biomedical advice, contributed interpretations and co-wrote the manuscript. All authors read and approved the final manuscript.

## Supplementary Material

Additional file 1 **Supplementary Data.** File format: PDF Size: 123K Click here for file
