# Supplementary material for: Coordinated modular functionality and prognostic potential of a heart failure biomarker-driven interaction network
Source: BMC Syst Biol. 2010 May 12;4:60. doi: 10.1186/1752-0509-4-60 (PMC2890499; doi:10.1186/1752-0509-4-60)
Supplement: Additional file 1 — Supplementary Data. File format: PDF Size: 123K [file 1752-0509-4-60-S1.PDF]

Coordinated modular functionality and prognostic potential of a heart failure biomarker-driven interaction network

Francisco Azuaje[1], Yvan Devaux[1] and Daniel R. Wagner[1,2]

[1] *Laboratory of Cardiovascular Research, Centre de Recherche Public - Santé, L-1150, Luxembourg*
[2] *Division of Cardiology, Centre Hospitalier, L-1210, Luxembourg*

Supplementary information

Table S1. List of HF biomarkers used as inputs to the PPI network inference and analysis framework

CRP	MMP2	AGT	ST2
TNF	MMP7	AVP	CHGA
IL1B	TIMP1	EDN1	CHGB
IL6	TIMP2	TNNI1	LGALS3
IL18	TIMP3	MYLK	TNFSF11
OLR1	TIMP4	FABP3	ADIPOQ
MPO	COL2A1	CKM	GDF15
MMP20	COL1A2	NPPB	
MMP9	PIIINP	NT-proBNP	
MMP3	REN	MR-proADM	

Table S2. List of candidate genes related to the set of HF biomarkers using the Endeavour software system. GSR: Global similarity ranking. The union of this list with the list shown in Table S1 represents the network seeds.

GSR	Gene	GSR	Gene	GSR	Gene
1	TIMP1	34	MPO	67	FBLN1
2	MMP2	35	COL3A1	68	COL5A2
3	IL6	36	COL6A1	69	SERPINA1
4	TIMP2	37	MMP20	70	ACAN
5	IL1B	38	MYLK	71	MMP16
6	MMP3	39	IL18	72	LPL
7	TNF	40	TNFSF11	73	IL6ST
8	MMP9	41	CKM	74	FBN1
9	COL1A2	42	TGFBI	75	APCS
10	TIMP3	43	MMP10	76	COL6A2
11	MMP7	44	COL5A1	77	LTA
12	ENSG00000206439	45	CRP	78	IL6R
13	ENSG00000206328	46	MMP17	79	PDGFRB
14	EDN1	47	VWF	80	COMP
15	AGT	48	IL1R1	81	COL8A1
16	NPPB	49	SPP1	82	ADAM15
17	COL1A1	50	ADAMTS1	83	THBS2
18	MMP1	51	GDF15	84	CCL2
19	REN	52	NPPA	85	FBLN2
20	COL6A3	53	IL1A	86	CYR61
21	CHGA	54	TGFB1	87	COL5A3
22	TIMP4	55	COL11A1	88	C3
23	ADIPOQ	56	LIF	89	DPT
24	MMP8	57	COL7A1	90	AVP
25	COL2A1	58	COL4A1	91	BMP2
26	SERPINE1	59	OLR1	92	C5
27	LGALS3	60	LAMC1	93	F12
28	COL4A2	61	COL10A1	94	IL1RAP
29	FN1	62	IL1RN	95	PDGFRA
30	MMP13	63	FABP3	96	LGALS3BP
31	MMP19	64	COL4A3	97	TNC
32	CHGB	65	SPARC	98	LAMB1
33	MMP14	66	ITGB1	99	ADAMTS5
				100	COL4A5

Table S3. Characteristics of the MI patients used for gene expression analyses.

		High EF (n=16)		Low EF (n=16)		*P*
1-month EF, % (median-range)		63	45-73	35	20-40	3.E-04
Age, y (median-range)		59	46-86	72	40-85	0.59
Sex (male, n, %)		13	81%	13	81%	1
Body Mass Index (median-range)		28	23-35	26	20-38	0.25
Cardiovascular history, n (%)						
	Prior MI	2	13%	3	19%	0.64
	CABG	0	0%	1	6%	0.33
	PTCA	1	6%	2	13%	0.56
	Diabetes	4	25%	6	38%	0.46
	Hypertension	6	38%	7	44%	0.73
	Hypercholesterolemia	7	44%	9	56%	0.50
	Tobacco	4	25%	5	31%	0.71
Medications, n (%)						
	Beta-blockers	16	100%	14	88%	0.16
	Calcium antagonists	3	19%	0	0%	0.08
	Nitrates	6	38%	6	38%	1
	ACE inhibitors	10	63%	10	63%	1
	Statins	14	88%	14	88%	1
	Angiotensin inhibitors	2	13%	0	0%	0.16

All patients had successful mechanical reperfusion and stenting of the infarct artery within 12 hours of chest pain onset. All patients received Aspirin, Clopidogrel, Heparin and Abciximab.

ACE: Angiotensin-Converting Enzyme; CABG: Coronary Artery Bypass Grafting; EF: Ejection Fraction; PTCA: Percutaneous Transluminal Coronary Angioplasty; MI: Myocardial Infarction.

Table S4. Description of network modules. BP and CC are examples of biological processes and cellular localizations terms respectively, which are highly over-represented in each module, as defined in the GO (January 2009 release). *P* values estimating the statistical significance, after correcting for multiple-testing, of the enrichment of the terms are also included. “*”: enrichment of GO term in the module was nominally significant at *P* = 0.05, i.e. no statistical significance was observed after correcting *P* value for multiple-testing. “?”: statistically detectable GO terms were not found.

**Module**	**Proteins**				**BP**	**CC**
1	CD46<br>C3<br>PFC<br>IF<br>CFHL5<br>CFHL3<br>CFHL4<br>ITGAX<br>BF	C3AR1<br>CFB<br>CFHR4<br>CFHR5<br>CFI<br>GC<br>GPR77<br>ITGAM<br>MASP1	OLFM4<br>PAPPA<br>CFHR3<br>CFP<br>CR1<br>CR2<br>CPN1<br>C5<br>C6	C7<br>C8B<br>C5R1<br>CPB2<br>C5AR1<br>C2	Innate immune response<br>(*P* = 2.0E-22)<br>Acute inflammatory response<br>(*P* = 4.4E-22)	Extracellular region part<br>(*P* = 5.1E-17)<br>Intracellular space<br>(*P* = 2.8E-7)
2	CRP<br>CFH<br>SNRP70<br>GP6<br>HIST1H1A	HIST1H2AL<br>LEP<br>spa<br>FCN2<br>APCS<br>C4BPA	FCGR3A<br>FCGR3B<br>CALU<br>TG<br>FCGR1A	FCGR2B	Immune response<br>(*P* = 5.0E-04)	Cell part<br>(*P* = 0.01)<br>Extracellular space (*P* = 0.05)
3	COL7A1<br>LAMB3<br>LAMC2<br>LAMA1<br>FBXL2	HSPA8<br>SHKBP1<br>ZC3H7A<br>LAMA5<br>LAMC1	SNAPAP<br>CGI-116<br>ATF7IP<br>CCDC53<br>COL6A3	PDIA3<br>BALF4<br>LAMB1<br>ACHE<br>ATXN7L2<br>GFI1B	Cell adhesion<br>(*P* = 1.8E-06)<br>Epithelial cell proliferation<br>(*P* = 6.0E-06)	Extracellular matrix part<br>(*P* = 8.4E-14)<br>Laminin complex<br>(*P* = 8.4E-14)
4	DPT<br>LOX<br>BAT3<br>CMA1<br>EDN1<br>COPS6<br>ADM<br>ECE1	EDNRB<br>KEL<br>EDNRA<br>MME<br>NPPA<br>EPB41<br>NPR3<br>NPR2	NPPB<br>EWSR1<br>NPR1<br>AGT<br>ACE2<br>AGTR2<br>CTSG<br>DNPEP	KNG1<br>MAS1<br>MEP1A<br>PRCP<br>PREP<br>PRG2<br>AGTR1<br>ENPEP<br>Agtr1	Circulation<br>(*P* = 2.3E-19)<br>Regulation of blood vessel size<br>(*P* = 2.7E-14)	Plasma membrane<br>(*P* = 0.003)<br>Intracellular<br>(*P* = 0.01)
5	LRP2<br>LPL<br>EMD<br>ASCC2<br>FLJ13855<br>RPL18A<br>KIAA1377<br>LUC7L2	PTPN4<br>VLDLR<br>CETP<br>APOC2<br>UBE2Z<br>TNFRSF11B<br>VWF<br>F8	TRA1<br>HSPA5<br>PDIA4<br>ADAMTS13<br>EBNA6<br>BILF1<br>q1hvi2_ebvg<br>BHRF1	ZNF512B<br>CALR<br>F12<br>APOH<br>KLKB1<br>MMP12<br>KRT1<br>GP1BA	Coagulation<br>(*P* = 1.6E-08)<br>Regulation of body fluids (*P* = 3.2E-08)	Extracellular region part<br>(*P* = 2.0E-04)<br>Chylomicron<br>(*P* = 2.0E-04)

Table S4 (cont.)

6	TGFB1	VTN	ITGB8	MGP	Tissue remodeling	Integral to plasma
	FNTA	FCN1	LTBP3	BMPR1B	(*P* = 8.4E-05)	membrane (*P* = 0.005)
	SLITL2	ACVRL1	LTBP4	ACVR1		
	SNIP1	YWHAE	VASN	ASCL1	Cell communication	Intracellular
	EIF3S2	TGFBRAP1	BMP2	BMPER	(*P* = 3.3E-09)	(*P* = 3.0E-05)
	FMOD	FKBP1A	BMPR1A	CHRDL2		
	TGFBR1	CCL3	BMPR2	GREM2		
	TGFBR3	ITGB6	SOSTDC1	NOG		
	TGFBR2	DAXX	ACTR2	ENG		
				TGFB2		
7	MYOC	COCH	TGFBI	MEP1B	Cell adhesion	Extracellular region
	COL1A2	PRELP	COL4A4	ITGB7	(*P* = 2.7E-18)	part (*P* = 9.9E-47)
	SHBG	BGN	FBLN2	REG3A		
	q69hr1_cioin	CHAD	BFLF2	TSHR	Anatomical structure	Collagen (*P* = 6.9E-33)
	CD36	COL9A2	FBN1	TRIB3	development	
	ITGA2B	COL9A3	FBN2	CTSD	(*P* = 9.8E-09)	
	ITGB3	LOXL4	MFAP2	GALNT6		
	ANXA5	TNFRSF10A	CSPG2	LRG1		
	COL10A1	MATN1	ELN	NT5E		
	P4HB	DCN	VCAN	SCGB1A1		
	COL2A1	COL4A1	HSPG2	TAC1		
	COL9A1	DISC1	FBLN1	FST		
	COMP	SMAD1	MPG	FSTL3		
	FGF7	SAA2	FGB	HGF		
	COL6A1	SAA4	ECM1	SMAD9		
	MAG	COL16A1	PREI3	CD79A		
	COL3A1	COL4A2	E6	NOV		
	PCOLCE	FAM46A	ATN1	C1QA		
	DDR1	ANTXR2	MFAP5	COL4A6		
	COL11A1	BMP3	NID	TMPRSS6		
	THBS1	COL4A3	NID1	COL6A2		
	COL5A1	CAMK2B	CTGF	ABCD3		
	SDC3	COL4A3BP	SMAD4	ADAMTS1		
	COL5A3	USH2A	LTBP1	FURIN		
	BMP1	SAA1	SKIL	VEGF		
	COL5A2	OSM	FN1	A2M		
	COL1A1	COL4A5	LACRT	VEGFA		
	TXN	RNF10	MIA	SPARC		
	NID2	HABP2	AMBP	CTSK		
	CAPN1	SERPINE2	VHL	PDGFB		
	DDR2	APP	LPA	COL13A1		
	HTRA1	CD93	GSN	PLAT		
	PKD1	MATN2	F13A1	SDC2		
				TGM2		

Table S4 (cont.)

9	SPP1	IL8	IGFBP3	CLDN1	Protein digestion	Extracellular region
	SGTA	BTC	BCAN	SPOCK1	(*P* = 2.6E-13)	part (*P* = 5.1E-33)
	PDLIM7	RECK	CCL7	TFPI		
	CTNNBL1	MMP7	MMP13	MMP8	Collagen metabolic	Extracellular space
	ABCF3	FASLG	ADAMTS5	UMOD	process	(*P* = 10.0E-19)
	ACP5	HBEGF	MMP14	TIMP2	(*P* = 6.1E-13)	
	PRKG1	MBP	LUM	SNCG		
	SERPINE1	NGFB	C1QBP	PSMA7		
	PLAU	CD44	ADI1	AGC1		
	ORM1	PLG	SDC1	MMP19		
	KLK2	CHGA	BCAR1	MMP20		
	LRP1B	SCG3	ACTB	TNFSF11		
	F2	THBS2	LRP1	KHDRBS1		
	IGFBP5	THBS3	MMP17	ADAM19		
	UBQLN4	TIMP1	KISS1	AKT1		
	ITGAV	RECQL5	MMP16	MAPK1		
	CYR61	ECH1	MMP2	MAPK8		
	ITGB5	EEF1B2	COL18A1	NFKB1		
	HAPLN1	MMP3	FGFR1	NFKBIA		
	MMP10	CCL8	BACE1	TNFRSF11A		
	MMP9	SPOCK3	CXCL12	TRAF6		
	CXCL5	MMP1	MMP25	TIMP3		
	CXCL6	BSG	PZP	EFEMP1		
	AREG	SERPINA3	LCN2	KDR		
	CXCL1	CCL13	TIMP4	ADAM17		
10	GEMIN4	CUBN	PHB2	MAP3K1	Positive regulation	?
	FCGR2A	CYHR1	CD14	IKBKE	of signal	
	C1GALT1C1	LIM2	PPIC	TRADD	transduction	
	CSNK1A1	SUFU	LMP2	MAP3K7IP2	(*P* = 1.3E-05)	
	CSNK2A1	LGALS1	NFKB2	TBK1		
	CSNK2A2	LGALS3BP	RELA			
11	ELA2	PRTN3	ADAMTS4	MMP11	Proteolysis	Endoplasmatic
	SERPINA1	CTRB1	DERL2	MMP26	(*P* = 2.4E-13)	reticulum
	KLK13	ELA1	DERL3	PRSS2		(*P* = 0.004)
	KLK3	PRSS1	KLK5	PRSS3	Digestion	
				try1_bovin	(*P* = 1.4E-07)	
				SSR1		
				CANX		
12	TNFRSF1A	POU2F1	IL18	MYD88	Immune response	Extracellular space
	LTA	ADAM9	IL18BP	TICAM2	(*P* = 7.1E-13)	(*P* = 0.002)
	LTB	IFNG	CASP3	IL1RAP		
	LGALS2	PDIK1L	IL18RAP	IRAK1		
	LTBR	VACCL3_190	IL1RL2	TIRAP	Apoptosis	
	TNFRSF14	crmE	CASP4	SIRPA	(*P* = 10.0E-05)	
	TUBA4A	IL1B	IL18R1	PRPF40A		
	TUBA1A	MAPK8IP2	IL1R1	RAC1		
	TNFRSF1B	ZNF675	SIGIRR	IL1R2		
	TNF	ADRB2	IRAK2	IL1RN		
	RALBP1	UBE2N	IL1F10	IL1A		
	TRAF2	CASP1	TOLLIP	NDN		
				S100A13		
				HAX1		
				NFKBIE		

Table S4 (cont.)

13	ITGA2<br>COL8A1<br>KLHL12<br>KRTAP4-12<br>EFEMP2<br>COL8A2<br>ITGA1<br>CD63<br>ITGB1<br>FLNA<br>ITGA3<br>RAB8B<br>ITGB1BP1<br>CD9<br>ILK<br>ITGA8<br>MCP	FLNB<br>GNB2L1<br>MAP4K4<br>ACTN1<br>TSPAN4<br>CD81<br>FLT4<br>CD151<br>ITGA9<br>FBXO2<br>CD47<br>TLN1<br>ITGA6<br>YWHAB<br>NME1<br>PXN<br>LGALS8	ITGA10<br>SLC3A2<br>TGOLN2<br>ICAM4<br>IGF1R<br>ACTN4<br>ARHGAP5<br>CD82<br>DOK1<br>EPS8<br>ITGA11<br>ITGA4<br>ITGB1BP3<br>NF2<br>PRKCE<br>PTK2<br>VCAM1	ITGB1BP2<br>Apba1<br>Tln1<br>Dab1<br>tenc1_human<br>ANKS1B<br>RABGAP1<br>RGS12<br>Shc1<br>GULP1<br>EMILIN1<br>CKM<br>TRIM63<br>UBC<br>CKB<br>MYOM2<br>FHL2	Cell adhesion<br>(*P* = 4.4E-15)<br><br>Cell matrix adhesion<br>(*P* = 2.4E-14)	Receptor complex<br>(*P* = 7.5E-12)<br><br>Integrim complex<br>(*P* = 1.2E-11)
14	ITGA5<br>TNC	CSPG3<br>EGFR	PTPRB<br>CNTN1	NCAN	Cell adhesion<br>(*P* = 2.0E-04)	?
15	MAPK3<br>MYLK<br>PRKG2<br>CALM1	CTTN<br>PAK1<br>MLC1<br>ACTC1	CAMK2G<br>PAK2<br>PRKCA<br>ACTA1	TNNI1<br>DIPA<br>PKD2L1<br>TNNC1<br>CCDC85B<br>TNNT1	Muscle contraction<br>(*P* = 5.1E-05)<br><br>Protein amino phosphorylation<br>(*P* = 10.0E-05)	Myofibril<br>(*P* = 9.0E-04)<br><br>Contractil fiber part (*P* = 9.0E-04)
16	SRC<br>IL6R<br>MAGI1<br>WWP1<br>WWP2<br>CNTF<br>STAT3<br>JAK1<br>IL6<br>PTHLH<br>ZBTB16<br>SH3GL2<br>IL6ST<br>LIFR<br>LIF<br>TLE1<br>AR<br>CNTFR<br>SOCS3<br>CDK9<br>ERBB2<br>ERBB3<br>OSMR<br>PRKCD<br>PTPN6<br>CTF1<br>IL31RA<br>PIK3CG	PLAUR<br>HCK<br>VAV1<br>JAK2<br>TYK2<br>SHC1<br>PTPN11<br>GRB2<br>ADAM15<br>SNX9<br>TRIP13<br>MAD2L2<br>LCK<br>MAD2L1<br>ABL1<br>PACSIN3<br>NPHP1<br>ArgBP2a<br>LYN<br>PTPN3<br>ARHGEF7<br>ARHGEF6<br>SH3PXD2A<br>SH3MD4<br>SH3PX3<br>SORBS1<br>q9ham2_human<br>SH3GLB1	FYN<br>YES1<br>NCK1<br>PDGFRB<br>VAV2<br>VAV3<br>COPA<br>NCK2<br>RAF1<br>SH3KBP1<br>EDG1<br>EIF2AK2<br>SNX1<br>RASA1<br>ARAF<br>GAB1<br>GRB7<br>BAG1<br>COPB1<br>GRB10<br>PDAP1<br>PDGFD<br>PTPN1<br>PTPN2<br>PTPRJ<br>PIK3CA<br>PIK3R2<br>PIK3R3	PRDX2<br>SOCS1<br>SYNGAP1<br>E5<br>ATP6V0C<br>Ptpn1<br>Ptpn11<br>SLC9A3R2<br>PDGFRA<br>PDGFA<br>PDGFC<br>CRKL<br>SNX6<br>SHB<br>SHF<br>CBL<br>CRK<br>PLCG1<br>SLC9A3R1<br>CAV1<br>CAV3<br>SNX2<br>SNX4<br>GRB14<br>STAT1<br>STAT5A<br>STAT5B<br>PIK3R1	Cell communication<br>(*P* = 7.7E-27)<br><br>Signal transduction<br>(*P* = 6.5E-23)	Cytoplasm<br>(*P* = 0.007)

Table S4 (cont.)

17	PTEN<br>CHGB<br>ACACA<br>CBFB<br>DKFZp761P0423<br>FLJ22471<br>GCS1<br>MARK3	MGLL<br>OGG1<br>PTK9L<br>SAFB2<br>ATXN2<br>TAZ<br>FGFR3<br>POLD1	S100A8<br>SLC25A6<br>YLPM1<br>TUBB2<br>CCDC92<br>POLR2E<br>RXRG<br>TWF2	UBTF<br>TUBB2A<br>SGK223<br>q8nbb9_human<br>hCG_1723909<br>OGG1 type 1e	Response to stress*<br>(*P* = 0.01)	DNA-directed RNA polymerase complex*<br>(*P* = 0.04

Software code.

Java-written routines for searching and retrieving interactions of biomarkers ("biomarkers.txt") from a (binary) protein-protein interaction text file (tab-separated, two-column).

```
/*
 * @author Francisco Azuaje
 */

package datamanager;
import java.io.*;
import java.util.*;
import java.lang.Math.*;

public class NewMain {

    public static int maxLines = 10000;
    public static int maxFeatures = 20;
    public static int maxInputBiomarkers = 800;
    static String [] biomarkers = new String[maxInputBiomarkers];
    static String [] numberProteinInteractions= new String[maxInputBiomarkers];
    static String [] retrievedGenes = new String[maxInputBiomarkers];
    static String [][] interactions = new String [maxLines][2];
    static String [][] retrievedInteractions= new String [maxLines][2];
    static String [][] retrievedPathways= new String [maxLines][maxFeatures];
    static String [][] GOresults = new String[38][6];
    static String [][] sample = new String[maxLines][maxFeatures];
    public static int numberSamples = 0;

    public static int numberBiomarkers = 0;
    public static int numInteractions = 0;

    static boolean  [] interactionRetrieved = new boolean [maxLines];
    public static int numberFeatures = 0;

   /**
     * @param args the command line arguments
     */

     public static void main(String[] args) {
        // TODO code application logic here
    }


     /** procedure for opening file with interaction data, and search/retrieval of interactions
     /* containing biomarkers stored in "biomarkers.txt"

     public static void fileReader(File inputFile) {
```

```
      int linecounter = 0;
      String str = new String("");
      String entry [] = new String[maxLines];
      BufferedReader in = null;

      if (inputFile.getName() != null) {

       try {
       in = new BufferedReader(new FileReader(inputFile));
       }

     catch (Exception e1) {
     e1.printStackTrace();
    }
   }

   try {
   while ( (str = in.readLine()) != null) {
   entry[linecounter] =  str;
   linecounter++;
   }
   numberSamples = linecounter;
   in.close();

   } //try

   catch (Exception e1) {
   e1.printStackTrace();
   }

   String tempTok = new String();

   for(int i=0; i<numberSamples; i++)
     {
     StringTokenizer st = new StringTokenizer(entry[i],"\t");
     int numberTok = 0;
     while (st.hasMoreTokens()) {
     tempTok = st.nextToken();
       if (!tempTok.equals(null))
       if((!tempTok.equals("\t") || (!tempTok.equals("\n")) || (!tempTok.equals(" "))))
       sample[i][numberTok] =  tempTok;
       numberTok++;
   }

 }

openBiomarkerFile();
searchInteractions();
```

```
  }

/* search/retrieval of interactions

            public static void searchInteractions()

            {
                numInteractions = 0;
                            for (int i = 0; i < numberBiomarkers; i++)
                                    {
                                                for (int j = 0; j < numberSamples; j++)
                                                {
                                                if((biomarkers[i].equals(sample[j][0])) ||
                                                (biomarkers[i].equals(sample[j][1])))

                                                            {
                                                            if(!interactionRetrieved[j])
                                                                        {
                                                                        retrievedInteractions[numInteractions][0]=
                                                                        sample[j][0];
                                                                        retrievedInteractions[numInteractions][1]=
                                                                        sample[j][1];
                                                                        interactionRetrieved[j]=true;
                                                                        numInteractions++;
                                                                        }
                                                            }

                                                } // for j

                        } // for i
  }


public static void saveRetrievedInteractions (File inputFile){

                        BufferedWriter out = null;
                        if (inputFile != null) {
                          try {
                              out = new BufferedWriter(new FileWriter(inputFile));
                              PrintWriter outputFile = new PrintWriter(out);
                              outputFile.println("num inter:" + numInteractions);
                              outputFile.println("num biomar:" + numberBiomarkers);
                              outputFile.println("num inter ori:" + numberSamples);
                             for (int i=0; i<numInteractions; i++)
                                    {
                                      for (int j=0; j<2; j++)
                                                {
                                                  if(retrievedInteractions[i][j]!= null)
                                                  outputFile.print(retrievedInteractions[i][j]);
```

```
                        Double value = new Double (sample[i][j]);
                        if (value.doubleValue() > 0.0)  outputFile.print(value*1000);
                        outputFile.print("\t");
                        }
                    outputFile.println();

                    }

                    outputFile.close();
                } //try

        catch (IOException e1) {
        e1.printStackTrace();
    }

  }

}

public static void openBiomarkerFile (){

     int linecounter = 0;
     String str = new String("");
     String entry [] = new String[maxLines];
     BufferedReader in = null;
     File inputFile = new File ("biomarkers.txt");
     if (inputFile.getName() != null) {
        try {
        in = new BufferedReader(new FileReader(inputFile));
        }
     catch (Exception e1) {
     e1.printStackTrace();
    }
   }

     try {
           while ( (str = in.readLine()) != null) {
           entry[linecounter] =  str;
           linecounter++;
           }
     numberBiomarkers=linecounter;
     in.close();

     } //try

    catch (Exception e1) {
    e1.printStackTrace();
    }

    String tempTok = new String();
```

```
    for(int i=0; i<numberBiomarkers; i++)
     {
        StringTokenizer st = new StringTokenizer(entry[i],"\t");
        int numberTok = 0;
        while (st.hasMoreTokens()) {
                tempTok = st.nextToken();
                if (!tempTok.equals(null))
                if((!tempTok.equals("\t") || (!tempTok.equals("\n")) || (!tempTok.equals(" "))))
               biomarkers[i] =  tempTok;
               numberTok++;
        }
      }

}

/* procedure for saving results

public static void fileSaver(File inputFile) {

        BufferedWriter out = null;
        if (inputFile != null) {
               try {
                    out = new BufferedWriter(new FileWriter(inputFile));
                    PrintWriter outputFile = new PrintWriter(out);

                    for (int i=0; i<numberSamples; i++)
                       {
                       for (int j=0; j<numberFeatures; j++)
                               {
                                 if(sample[i][j]!= null)
                                 outputFile.print(sample[i][j]);
                                if (value.doubleValue() > 0.0)  outputFile.print(value*1000);
                                 outputFile.print("\t");
                               }
                     outputFile.println();

                       }
                    outputFile.close();

       } //try

        catch (IOException e1) {
        e1.printStackTrace();
   }

 }


*/
```

```
    saveRetrievedInteractions(inputFile);
    }
}
```
